# The Association between Mental Health Symptoms and Quality and Safety of Patient Care before and during COVID-19 among Canadian Nurses

**DOI:** 10.3390/healthcare10020314

**Published:** 2022-02-07

**Authors:** Farinaz Havaei, Xuyan Tang, Peter Smith, Sheila A. Boamah, Caroline Frankfurter

**Affiliations:** 1 School of Nursing, University of British Columbia, Vancouver, BC V6T 2B5, Canada; caroline.frankfurter@ubc.ca; 2 Department of Educational and Counselling Psychology, and Special Education, University of British Columbia, Vancouver, BC V6T 1Z4, Canada; xuyan.tang@ubc.ca; 3 Dalla Lana School of Public Health, University of Toronto, Toronto, ON M5T 3M7, Canada; psmith@iwh.on.ca; 4 The Institute for Work & Health, Toronto, ON M5G 1S5, Canada; 5 School of Nursing, McMaster University, Hamilton, ON L8S 4K1, Canada; boamahs@mcmaster.ca

**Keywords:** nurses, care delivery, mental health, quality and safety, COVID-19

## Abstract

(1) Background: While the association between nurse mental health and quality and safety of patient care delivery was well documented pre-pandemic, fewer research studies have examined this relationship in the context of COVID-19. This study examines the impact of various mental health symptoms experienced by nurses on quality and safety before and during the COVID-19 pandemic; (2) Methods: A secondary analysis of cross-sectional survey data from 4729 and 3585 nurses in one Canadian province between December 2019 and June-July 2020 was conducted. Data were analyzed using between group difference tests and logistic regression; (3) Results: Compared to pre-COVID-19, during COVID-19 nurses reported a higher safety grade, a greater likelihood of recommending their units for care and lower quality of nursing care. Most mental health symptoms were higher during COVID-19 and higher levels of mental health symptoms were correlated with lower ratings of quality and safety both pre- and during COVID-19; (4) Conclusion: Mental health symptoms have implications for nurses’ quality and safety of patient care delivery, with the association between mental health symptoms and quality and safety following a dose–response relationship before and during COVID-19. These findings suggest that it is worthwhile for nurse mental health symptoms to be included as hospital level performance metrics.

## 1. Introduction

Nursing is one of the most stressful occupations in the world [1]. Nurses face high-stress situations in the workplace on a regular basis, including heavy workloads, insufficient staffing, team conflict, and witnessing patient suffering and death [2,3]. In the face of workplace stressors, nurses are at an increased risk of mental health problems [2,3]. The impact of nurses’ mental health on their work behaviours (e.g., absenteeism [4], presenteeism [5] and their quality and safety of patient care provisions) were well documented pre-pandemic [6,7]. However, less research has examined these associations in the context of COVID-19. This is particularly important as nurses play a major role in contributing to or preventing the 136 million patient adverse events and the associated 2.6 million patient mortalities per year worldwide [8]. Given the deterioration of nurses’ mental health during COVID-19 [9,10,11,12,13] and an increasing shortage of nurses worldwide [14,15], there is an urgent need to re-evaluate and better understand the relationship between nurse reported mental health and perceived quality and safety of patient care. The primary purpose of this study is to investigate the association between the severity of specific mental health symptoms, including anxiety, depression, post-traumatic stress disorder (PTSD) and three burnout dimensions (emotional exhaustion, depersonalization and personal accomplishment) and their unique impact on nurses’ reports of quality and safe patient care delivery pre- and during COVID-19. The secondary aim is to evaluate how nurses’ reports of mental health, quality and safety have changed during COVID-19. The findings will shed light on specific mental health problems that most strongly and consistently compromise nurses’ provision of quality and safe care and offer potential mental health strategies for this workforce.

Since COVID-19, there has been a rise in mental health problems among healthcare workers including nurses. Several meta-analyses and systematic reviews examined the impact of COVID-19 on healthcare worker’s mental health. Among them, a 2021 meta-analysis of 70 high quality studies across 23 countries and 3 continents showed a high prevalence of sleep disorders (44%), depression (31.1%), anxiety (30%) and PTSD (20.2%) among healthcare workers during COVID-19 [11]. Another systematic review of 16 nurse burnout studies during COVID-19 estimated the prevalence ranging from 12.6% (for depersonalization) to 34% (for emotional exhaustion) [13]. In Canada, a recent study using data from three time points demonstrated an increasing prevalence of anxiety and depression by 10% to 15% among nurses between December 2019 (anxiety = 31%; depression = 20%) and April/May of 2020 (anxiety = 45%, depression = 31%), with levels remaining high when measured again in June/July 2020 (anxiety = 43%, depression = 30%) [10].

Pre-pandemic research has examined the impact of nurse mental health on their quality and safety of patient care delivery with most studies focusing on burnout. A systematic review of studies published up to July 2015 identified 46 studies across 16 countries and 6 continents. Of these studies 19 focused solely on burnout, 16 studies on other mental health problems and 11 studies included both [6]. The same systematic review concluded poor mental health (e.g., depression and anxiety), including burnout, was associated with lower quality and safe patient care delivery among healthcare workers, mostly composed of nurses [6]. In most studies, poor quality and safety of patient care was operationalized through healthcare workers’ reports of patient adverse events and mental health symptoms through their absence or presence rather than their severities [6]. Another systematic review of 102 studies over a similar time period (until March 2015), with over 210,000 healthcare providers (52% nurses) across 32 countries and 6 continents, estimated small to medium sized relationships between burnout and lower quality and safety of patient care delivery, also mostly operationalized through self-reports [7]. The systematic review found the strongest link belonged to emotional exhaustion and quality and safe care delivery. While these studies suggest that mental health symptoms are associated with quality and safety of patient care, it is important to re-examine this relationship within the context of the large increases in the prevalence of mental health symptoms, during the pandemic. It is equally important to examine how nurse mental health symptoms and quality and safe care provision changed during the pandemic in light of increasing measures of infection prevention and control and a growing shortage of nursing staff [14,15].

## 2. Materials and Methods

### 2.1. Data Collection and Sample

This study is a secondary analysis of data from two provincial cross-sectional surveys conducted through a partnership between university nursing researchers and the British Columbia Nurses’ Union (BCNU). The BCNU represents approximately 48,000 nurse members in the province of British Columbia, Canada. The first survey was conducted in December 2019 (Time 1, pre-COVID-19) and the second survey in June 2020 (Time 2, during COVID-19). At both times, the BCNU sent out an email invite to its nurse members asking them to complete the 25 min electronic survey. During the four-week data collection period, multiple strategies, such as weekly email reminders, social media advertisement and a raffle draw, were used to increase recruitment. Respondents were informed of the voluntary and confidential nature of their participation and that a survey submission would imply consent.

The total number of returned surveys was 5512 in Time 1 and 4523 in Time 2, yielding a response rate of approximately 12% and 10%, respectively. Comparative analyses of both study samples with the provincial and national nursing workforce revealed no large differences in demographics. For this study, only responses from actively working registered nurses (RN) and/or registered psychiatric nurses (RPN) and licensed practical nurses (LPN) were included in our analysis, yielding samples that involved 4729 and 3585 of nurses from Times 1 and 2 surveys, respectively. A priori power calculation showed a sample size of 403 nurses would be sufficient to detect small effect sizes in logistic regression analysis with 1 predictor at alpha = 0.05. Ethics approval was obtained from the University’s Behavioural Research Ethics Board at both survey times (Time 1: H18-02724; Time 2: H20-01861).

### 2.2. Measures

#### 2.2.1. Outcomes

Quality and safety of patient care delivery was measured using 3 questions from the international RN4CAST, a 12-country study of nurses’ work environment conditions and their impact on quality and safety [16,17,18,19]. Questions asked nurses to: give their primary unit an overall safety grade on a five-point scale ranging from ‘failing’ to ‘excellent’; rate the general quality of nursing care they delivered to patients on their unit using a four-point response scale with options ranging from ‘poor’ to ‘excellent’; and indicate their likelihood of recommending their unit to friends and family for care on a four-point scale ranging from ‘definitely no’ to ‘definitely yes’. Higher scores represented higher ratings of quality and safety. For logistic regression analysis, responses to the three questions were dichotomized and reverse scored: high (0) and low (1) quality and safe patient care. Similar to previous research, this study used these quality and safety questions as individual outcomes.

#### 2.2.2. Predictors

Mental health predictors included anxiety, depression, PTSD and burnout. Anxiety was measured using the seven-item General Anxiety Disorder Scale (GAD-7) with the following established diagnostic criteria: 0–4 (none), 5–9 (mild), 10–14 (moderate), and 15–21 (severe) [20]. Depression was measured using the nine-item Patient Health Questionnaire (PHQ-9) with the following cut-offs: 0–4 (none), 5–9 (mild), 10–14 (moderate), 15–19 (moderately severe), and 20–27 (severe) [21]. PTSD was measured by the Post-Traumatic Stress Syndrome 14 Questions Inventory (PTSS-14) with a cut-off score of 45 or higher as a positive screen for PTSD [22]. Burnout was measured by the 22 item Maslach Burnout Inventory-Human Services Survey (MBI-HSS) with three subscales: Emotional Exhaustion (EE, nine items), Depersonalization (DP, five items) and Personal Accomplishment (PA, eight items) (acronyms used in the Methods and Results Sections only) [23]. Cut-off criteria were applied to each subscale: for EE, scores 0–16 (low), 17–26 (moderate), and 27–54 (high); for DP, scores 0–6 (low), 7–12 (moderate), and 13–30 (high); and for PA, scores 0–31 (low), 32–38 (moderate), and 39–48 (high) [23]. The PA subscale was reverse scored for analyses, such that higher scores reflected higher levels of burnout (i.e., reduced PA). A psychometric examination of the scales’ scores replicated a unidimensional factor structure for GAD-7, PHQ-9 and PTSS-14 at both survey times with excellent internal consistencies (Time 1 α: 0.90–0.93; Time 2 α: 0.90–0.94). For MBI-HSS, a three-factor structure was confirmed in both surveys with excellent total and subscale internal consistencies (Time 1: subscale α = 0.77–0.92, total scale α = 0.83; Time 2: subscale α = 0.78–0.93, total scale α = 0.82).

#### 2.2.3. Control Variables

Control variables included individual and workplace characteristics. Individual characteristics included gender, age, nursing experience, professional designation (LPNs, RPNs, RNs, dually registered as RPN and RN), education (diploma/certificate, undergraduate degree, graduate degree), employment status (full-time, part-time, casual) and role (direct care provider, nurse leader, educator). Workplace characteristics were healthcare sector (acute care, community care, long-term care) and geographical region (urban, suburban, rural). Among these variables, two individual characteristics were recoded into binaries: nurse education (0 = diploma/certificate, 1 = undergraduate and graduate degree) and nurse designation (0 = LPNs, 1 = RNs, RPNs, and dually registered).

### 2.3. Statistical Analysis

Data were analyzed using descriptive statistics and between-group difference tests in the Statistical Package for Social Sciences 27 (SPSS Inc., Chicago, IL, USA) and using logistic regressions with the R language (V.4.0.4 R Foundation, Vienna, Austria). Descriptive statistics were used to describe the two samples. Because Times 1 and 2 surveys were not linked over time, Pearson chi-square (χ^2^) test and independent samples t-test were used to examine potential differences in demographics across the two samples. The results would determine whether data from the two samples should be aggregated or analyzed separately. For example, sample heterogeneity would require an independent examination of data from each sample. To evaluate changes in nurses’ reports of quality and safety and mental health symptoms across the two times, an analysis of covariance (ANCOVA) was conducted after controlling for individual and workplace characteristics. Finally, assuming sample heterogeneity, logistic regressions, using pairwise deletion, were conducted with Times 1 and 2 samples separately. Because mental health variables were highly correlated, quality and safety outcomes were regressed on one mental health predictor per model resulting in a total of 18 regression models in each time point. Various severities of mental health symptoms were compared against their absence or low levels (reference group) using odds ratios and confidence intervals.

## 3. Results

### 3.1. Descriptive Findings, Between-Group Differences

Table 1 provides sample descriptions at each time point. Overall, in both surveys, most respondents were female, registered nurses (RN and/or RPN), with a nursing degree, held a full-time direct nursing care role and employed in urban, acute care settings. On average, respondents were slightly older than 40 with nearly half of them having fewer than 10 years of nursing experience.

Table 1 also shows between-group difference test results. With the exception of employment status, differences were found in other individual and workplace characteristics across Times 1 and 2 samples, suggesting sample heterogeneity. Compared to pre-COVID respondents, a slightly smaller proportion of the COVID sample self-identified as male (6% vs. 8%), registered nurse (RN and/or RPN) (81% vs. 84%), less experienced, with a degree (66% vs. 69%), a direct care provider (86% vs. 90%) and in acute care (63% vs. 74%) and rural (16% vs. 20%) areas. The COVID respondents were also older than pre-COVID respondents (43 vs. 41 years of age).

Table 2 shows descriptive statistics and comparisons of nurses’ ratings of their mental health, quality and safety across Times 1 and 2 after considering control variables. Compared to pre-COVID, nurses reported significantly higher anxiety (MT1 = 6.9, MT2 = 8.7, *p* < 0.001), depression (MT1 = 7.4, MT2 = 9.1, *p* < 0.001), PTSD (MT1 = 44.5, MT2 = 47.2, *p* < 0.001), and EE (MT1 = 28.2, MT2 = 30.4, *p* < 0.001) scores during COVID-19, with no statistically significant changes in DP and PA scores across the two time points. Table S1, included as Supplementary Material, demonstrates the proportion of various severities of each mental health symptom at each survey time. Nurses’ ratings of their unit’s safety grade (MT1 = 3.3, MT2 = 3.4, *p* < 0.001) and the likelihood of recommending their unit to family and friends for care (MT1 = 3.0, MT2 = 3.07, *p* < 0.001) increased slightly from pre-COVID to during COVID-19 (Table 2). Conversely, compared to pre-COVID, respondents reported lower ratings of quality of nursing care (MT1 = 3.24, MT2 = 3.17, *p* < 0.001) during COVID-19 (Table 2).

### 3.2. Results of Logistic Regression Analyses

Table 3 displays the results from logistic regression models regressing three quality safety outcomes on each mental health symptom after controlling for individual and workplace characteristics pre- and during COVID-19. The Hosmer–Lemeshow tests were non-significant suggesting good fitting models. Overall, we found that, compared to no or low levels, higher severities of each mental health symptom were associated with lower ratings of quality and safety at both survey times.

Before COVID-19, nurses with mild-to-severe anxiety were 2.2 (95% CI 1.9 to 2.5) to 4.3 (95% CI 3.4 to 5.5) times more likely to give lower quality and safety ratings compared to their peers who screened negative for anxiety symptoms. Our findings were similar during COVID-19, with odd ratios ranging from 1.9 (95% CI 1.6 to 2.3) to 3.4 (95% CI 2.7 to 4.3). Of note is the increasing pattern of odd ratios for increasing severity of mental health symptoms. For example, compared to nurses who screened negative for anxiety, nurses with mild anxiety were 1.9 (95% CI 1.6 to 2.3) times more likely to give a lower safety grade during COVID-19. The odd ratios increased to 3.1 (95% CI 2.5 to 3.8) and 3.4 (95% CI 2.7 to 4.3) for nurses with moderate and severe anxiety, respectively.

The findings were similar for depression and PTSD. Nurses with mild-to-severe depression were 1.8 (95% CI 1.5 to 2.2) to 6.8 (95% CI 5.0 to 9.4) times more likely to give lower ratings of quality and safety before COVID-19 and 1.5 (95% CI 1.2 to 2.0) to 7.2 (95% CI 5.1 to 10.2) times more likely to give low ratings during COVID-19. For PTSD, odd ratios ranged between 2.7 (95% CI 2.3 to 3.0) to 3.6 (95% CI 3.1 to 4.2) before COVID-19 and 2.4 (95% CI 2.1 to 2.8) to 2.7 (95% CI 2.2 to 3.4) during COVID-19. Consistent with anxiety, higher severities of depression were associated with lower quality and safety ratings before and during COVID-19.

For burnout, higher levels of the three domains were associated with lower quality and safety ratings. Specifically, nurses with moderate to high EE were 2.3 (95% CI 1.9 to 2.8) to 7.8 (95% CI 5.8 to 10.6) times more likely to give lower ratings of quality and safety before COVID-19 and 1.2 (95% CI 0.8 to 1.9) to 6.0 (95% CI 3.9 to 9.7) times more likely during COVID-19. One exception is, during COVID-19, the odd of not recommending the unit to family and friends for care was not statistically significant for nurses with moderate EE compared to their peers with low EE. Nurses with moderate EE were 2.5 (95% CI 1.8 to 3.6) times more likely than their peers with low EE to not recommend their unit to family and friends pre-COVID-19.

For the other two burnout domains, nurses with moderate-to-high levels of DP were 2.0 (95% CI 1.7 to 2.3) to 9.3 (95% CI 6.9 to 12.8) times more likely to give lower quality and safety ratings compared to their peers with low DP pre COVID-19 and 1.5 (95% CI 1.1 to 2.1) to 5.2 (95% CI 4.0 to 6.7) times more likely during COVID-19. For PA, odd ratios ranged between 1.6 (95% CI 1.4 to 1.9) and 4.4 (95% CI 3.3 to 5.9) pre-COVID-19 and 1.5 (95% CI 1.1 to 1.9) to 5.5 (95% CI 4.1 to 7.5) during COVID-19. Similar to all mental health predictors, higher levels of the three burnout subscales were associated with lower quality and safety ratings.

Figures S1–S6 (Supplementary Material) offer descriptive visual depictions of the relationships between various severities of each mental health symptom and quality and safety outcomes pre- and during COVID-19. No consistent patterns of changing relationships were found across the two time points. For example, while odd ratios reflective of the relationship between severe depression and safety grade decreased from Time 1 to Time 2, odd ratios representative of the association between severe depression and the other two quality and safety outcomes increased during the same time interval.

## 4. Discussion

This study had several key findings. First, compared to pre-COVID-19, nurses reported greater levels of most mental health symptoms during COVID-19. Second, while nurses gave their units a higher safety grade and reported a greater likelihood of recommending their units to family and friends for care, they provided lower ratings of quality of nursing care during COVID-19. Third, higher ratings of mental health symptoms were consistently associated with lower ratings of quality and safety during both time points.

With the exception of depersonalization and personal accomplishment, nurses’ reports of other mental health symptoms increased during COVID-19. This finding is in line with other international reports that had similarly suggested an increasing prevalence of mental health problems among healthcare workers, particularly nurses [10,11,12,13]. The stability of depersonalization and personalization may be attributed to greater levels of acknowledgement that nurses and other providers received from their organizations and the general public during COVID-19. Several campaigns were initiated to support healthcare workers around the world including their celebration through nightly applause and cheers [24]. It is highly likely that such initiatives created both a sense of community and feelings of reward and recognition, protecting nurses against experiences of depersonalization and personal accomplishment in the context of the pandemic. According to burnout experts, organizational interventions that alleviate employee depersonalization and low personal accomplishment must focus on building a sense of community and acknowledging job well done, respectively, as opposed to workload management strategies for alleviating employee emotional exhaustion [25].

In addition to rising reports of mental health symptoms, nurses had higher ratings of unit safety during COVID-19. This unsurprising finding is attributed to more strict infection prevention and control procedures adopted by healthcare settings to mitigate the risk of spreading COVID-19 infections. A systematic review of 61 studies accounting for nearly 300,000 employees, including healthcare workers across 3 continents, showed better infection prevention and control procedures in the workplace would reduce transmission of COVID-19, hence likely resulting in increased workplace safety perceptions [26].

An unanticipated finding was the increased likelihood of recommending units to family and friends for care during COVID-19. Previous research showed nurses were fearful of contracting COVID-19 and spreading it to their loved ones at home [27]. It is however possible that nurses’ higher likelihood of unit recommendation was also a result of more strict infection prevention and control measures, and hence greater safety perceptions during the pandemic. In the context of a highly infectious pandemic, the recommendation question was likely interpreted as implying an urgent case of care. In such critical circumstances, it is possible that nurses would highly recommend their unit for care in light of the higher safety precautions adopted by their healthcare settings.

Compared to the pre-pandemic period, nurses gave lower ratings of quality of nursing care during the pandemic. This finding likely reflects the increasing workloads nurses faced in the context of the pandemic. A discrete event simulation study recently observed that, compared to pre-COVID estimates, walking distance at work, mental workload, missed care, missed care delivery time and care task waiting time have all increased between 40% and over 300% among Canadian nurses during COVID-19 [28]. It is possible that, due to increasing workloads, nurses prioritized the medical/physical care of patients over their psychosocial care [29].

It is important to note that differences in nurses’ reports of mental health symptoms and quality and safety outcomes could also be a result of statistically significant differences in demographics of the two study samples (preCOVID-19 and COVID-19). However, given the small magnitude of most of these differences (2% to 4%), they are likely clinically insignificant. A more prominent difference between the two samples, however, was the occurrence of the COVID-19 pandemic, which perhaps was a more important contributing factor to some of the observed differences in the mental health symptoms and quality and safety ratings described above.

Consistent with previous research [6,7], we found poor mental health was associated with lower quality and safety ratings both pre- and during COVID-19. This association followed a dose–response relationship where more severe mental health symptoms were correlated with consecutively lower ratings of quality and safety. Previous research in this area has mostly evaluated the presence or absence of mental health symptoms rather than examining their severity [6,7,30]. However, given the dose–response relationship found in our study, it is likely that the binary operationalization of mental health symptoms would result in underestimated effects in relation to quality and safety.

This study also shed light on the most important mental health predictors of quality and safety before and during COVID-19. While quality and safety ratings were most strongly associated with emotional exhaustion and depersonalization pre-pandemic, these ratings were mostly influenced by emotional exhaustion and depression during the pandemic. This finding points to the importance that a nurse’s emotional exhaustion is one of the most important and consistent predictors of nurses’ quality and safety reports, given its stability across survey periods. This is congruent with previous research that identified nurse emotional exhaustion as the most important domain of burnout for predicting negative patient outcomes [31]. Of note is that moderate emotional exhaustion became a non-significant predictor of nurses’ likelihood of recommending their units for care during the pandemic. We believe this finding could be explained by the increasing attention nurses received and increasing safety measures introduced in healthcare settings during COVID-19, which likely buffered against moderately exhausted nurses not recommending their units to family and friends.

### 4.1. Implications

The increasing level of mental health symptoms during COVID-19 and their dose–response relationship with nurses’ reports of quality and safety point to an urgent need for healthcare policies that better prevent, detect and treat mental ill health among nurses.

In response to these mental health challenges, the first International Standard for Workplace Psychological health and Safety was finally published by the International Organization for Standardization (ISO), an independent non-governmental organization made up of members from the national standards bodies of 165 countries. The ISO provides a framework for identifying and addressing workplace risk factors to employee psychological health and safety across a wide range of industries and sectors including healthcare. In Canada, studies of the Canadian Standard identified workplace risk factors most predictive of nurses’ mental ill health and their quality and safety of care delivery [19,32]. Despite this progress, the implementation of ISO is yet to be mandated in healthcare by governments internationally.

In addition to the workplace risk factors identified in the ISO, other pandemic-specific workplace risk factors have also been found to influence nurse mental health. Examples include working in high-risk environments, caring for COVID-19 patients and adequate access to high quality personal protective equipment [33]. Beyond these factors, a recent study argued that timely policy measures would be more effective than any other workplace measures in early prevention and control of the pandemic and the associated mental health impacts [34]. These findings provide decision makers with directions to better protect the mental health of the nursing workforce during a highly contagious epidemic.

Mental health risk factors may also be non-work related. Pandemic research with healthcare workers found individual-level factors, such as personality [35,36], limited social support [36], and having chronically ill loved ones [33], are important mental health risk (or protective) factors. Contrary to work-related factors, individual-level risk factors are non-modifiable for the most part. Therefore, the onus is mostly on healthcare leaders and policy makers who could improve nurses’ working conditions, and on nursing education systems who could teach future nurses protective strategies, such as teamwork and crisis leadership skills [37].

Our study findings, particularly the presence of a dose–response relationship, also suggest that the early detection of mental health symptoms and their severities would be key to addressing potential or actual quality and safety issues in practice. Early detection requires confidential assessments of nurse mental health on a regular basis using standardized and validated measurement tools that may be used for benchmarking purposes. Our study findings provided preliminary evidence supporting the inclusion of nurse mental health as a hospital level performance metric given its consistent relationship with quality and safe patient care before and during COVID-19. We also strongly advocate for the public reporting of aggregated nurse mental health data along with other internationally known hospital performance metrics. Public reporting of nurse mental health will provide opportunities not only for benchmarking and quality improvement purposes, but also for keeping healthcare organizations and their operators accountable to the health and safety needs of their nursing workforce and those under their care [38,39]. Despite these nurse-specific recommendations, we acknowledge that other healthcare providers and their care provision would also likely benefit from similar strategies. To confirm, future research should determine the association among healthcare providers using more sophisticated research methods.

### 4.2. Strengths and Limitations

This study provides a novel examination of the association between mental health symptom severity and quality and safety before and during COVID-19. Despite this strength, we acknowledge several study limitations. First, the findings should be cautiously generalized to other samples. Our study used secondary data from nurses of one Canadian province who may not be representative of nurses in other contexts. Similarly, even the samples’ representativeness of the provincial nursing workforce may be questionable due to low response rates. Although comparative analyses revealed no large differences in samples’ demographics and the provincial and national nursing workforce [19,27], we still believe generalizing the results beyond the study sample should be conducted cautiously. Second, differences in nurses’ mental health symptoms and quality and safety ratings across the two surveys could potentially be a result of small differences in sample demographics (2–4%). That said, we believe, compared to the small demographic differences, the occurrence of the COVID-19 pandemic was a more prominent difference and likely the contributing factor to differences in nurse mental health symptoms and quality and safety ratings across the two study samples. Third, the study data originated from nurses’ reports, which have been criticized as a questionable approach for measuring quality and safety. However, previous research linked nurses’ reports of excellent quality of care with lower odds of negative patient outcomes assessed using administrative data, concluding nurses’ reports of quality of care as a valid indicator of healthcare performance [40]. Finally, the cross-sectional nature of the surveys does not allow any cause-and-effect conclusions to be made. We recommend future research to use longitudinal designs, multiple sources and types of data to establish the link between healthcare providers’ mental health and their quality and safety of care provision.

## 5. Conclusions

The bottom line of this research is that quality and safe patient care provision would not be possible without a healthy nursing workforce. Our study findings used data from pre- and during COVID-19 to demonstrate that, when nurses suffer, those under their care are also more likely to suffer. We also found more severe mental health symptoms have greater consequences for patient care provision. Given these findings, nurse mental health should be treated as a hospital level performance metric that is confidentially assessed, tracked over time and publicly reported.

## Figures and Tables

**Table 1:** Sample demographics before and during COVID-19.

Characteristics (Categorical Variables)	Before COVID-19	During COVID-19	*p*-Value ^a^
	*n* (%)	*n* (%)	
Gender	*n* = 4729	*n* = 3585	<0.001
Female	4331 (91.6)	3356 (93.6)	
Male	398 (8.4)	229 (6.4)	
Professional designation	*n* = 4729	*n* = 3585	<0.001
LPNs	757 (16)	675 (18.8)	
RNs, RPNs, and dually registered	3972 (84)	2910 (81.2)	
Education	*n* = 4729	*n* = 3585	0.001
Diploma/certificate	1459 (30.9)	1225 (34.2)	
Undergraduate and graduate degree	3270 (69.1)	2360 (65.8)	
Employment status	*n* = 4728	*n* = 3585	0.231
Full-time	2962 (62.6)	2209 (61.6)	
Part-time	1277 (27)	1026 (28.6)	
Casual	489 (10.3)	350 (9.8)	
Role	*n* = 4729	*n* = 3585	<0.001
Direct care provider	4231 (89.5)	3087 (86.1)	
Nurse leader	381 (8.1)	378 (10.5)	
Educator	117 (2.5)	120 (3.3)	
Healthcare sector	*n* = 4723	*n* = 3581	<0.001
Acute care	3480 (73.7)	2264 (63.2)	
Community care	822 (17.4)	856 (23.9)	
Long-term care	421 (8.9)	461 (12.9)	
Geographical region	*n* = 4709	*n* = 3568	<0.001
Urban	2953 (62.7)	2269 (63.6)	
Suburban	831 (17.6)	716 (20.1)	
Rural	925 (19.6)	583 (16.3)	
Nursing experience	*n* = 4714	*n* = 3570	<0.001
5 years or less	1416 (30.0)	862 (24.1)	
6 to 10 years	1006 (21.3)	711 (19.9)	
11 to 15 years	804 (17.1)	631 (17.7)	
16 to 20 years	392 (8.3)	359 (10.1)	
21 years or more	1096 (23.2)	1007 (28.2)	
Characteristics (Continuous Variables)	Before COVID-19	During COVID-19	*p*-Value ^b^
	Mean (SD)	Mean (SD)	
Age	*n* = 470,040.50 (11.60)	*n* = 355,342.57 (11.68)	<0.001

Note: ^a^ Pearson test; ^b^ Independent samples t-test.

**Table 2:** Comparisons of mental health symptoms and quality safety ratings before and during COVID-19.

	Pre-COVID-19 Responses		COVID-19 Responses		ANCOVA
	*n*	Mean (SD)	*n*	Mean (SD)	
Mental Health					
Anxiety	4241	6.94 (0.09)	3257	8.66 (0.10)	F (*1, 7487*) = 173.50, *p* < 0.001
Depression	4240	7.44 (0.09)	3237	9.07 (0.11)	F (*1, 7466*) = 132.55, *p* < 0.001
PTSD	4267	44.54 (0.29)	3243	47.16 (0.33)	F (*1, 7499*) = 36.12, *p* < 0.001
EE	4116	28.18 (0.20)	3151	30.41 (0.23)	F (*1, 7256*) = 53.44, *p* < 0.001
DP	4119	8.92 (0.10)	3153	8.82 (0.12	F (*1, 7261*) = 0.44, *p* = 0.51
PA	4073	13.64 (0.12)	3097	13.75 (0.14)	F (*1, 7159*) = 0.31, *p* = 0.58
Quality and Safety					
Safety grade	4096	3.28 (0.02)	3174	3.40 (0.02)	F (*1, 7259*) = 30.78, *p* < 0.001
General quality of nursing care	4095	3.24 (0.01)	3178	3.17 (0.01)	F (*1, 7262*) = 21.54, *p* < 0.001
Recommend to family and friends	4089	3.02 (0.01)	3169	3.07 (0.02)	F (*1, 7247*) = 6.40, *p* < 0.05

Note: ANCOVA, analysis of covariance; PTSD, post-traumatic stress disorder; EE, emotional exhaustion; DP, depersonalization; PA, personal accomplishment. The personal accomplishment subscale was reverse scored. For each mental health variable, total scores were used for the analysis with higher scores representing more severe mental health problems. Quality and safety variables were rated on a 4- or 5-point response scale with higher scores reflecting higher quality and safety. Individual and workplace characteristics were taken into account.

**Table 3:** Mental health symptoms severity as predictors of nursing quality and safe patient care delivery before and during COVID-19.

	Pre-COVID-19			During COVID-19		
	Safety Grade	General Quality	Recommend to Family and Friends	Safety Grade	General Quality	Recommend to Family and Friends
	OR (95% CI)	OR (95% CI)	OR (95% CI)	OR (95% CI)	OR (95% CI)	OR (95% CI)
Mild Anxiety ^a^	2.16 *** (1.86–2.51)	2.37 *** (1.81–3.13)	2.40 *** (1.97–2.94)	1.86 *** (1.55–2.25)	2.06 *** (1.47–2.94)	1.73 *** (1.31–2.30)
Moderate Anxiety ^a^	2.46 *** (2.03–2.98)	3.32 *** (2.45–4.52)	3.41 *** (2.71–4.31)	3.08 *** (2.47–3.84)	3.52 *** (2.47–5.09)	2.68 *** (1.99–3.62)
Severe Anxiety ^a^	4.30 *** (3.41–5.46)	3.97 *** (2.88–5.48)	6.36 *** (5.00–8.11)	3.38 *** (2.69–4.26)	5.41 *** (3.83–7.76)	4.58 *** (3.44–6.16)
Model=	8.55, *p* = 0.38	8.26, *p* = 0.41	13.29, *p* = 0.10	7.82, *p* = 0.45	15.68, *p* = 0.05	1.95, *p* = 0.98
*n*	4095	4094	4088	3126	3130	3122
Mild Depression ^b^	1.90 *** (1.63–2.21)	1.85 *** (1.41–2.44)	1.78 *** (1.45–2.18)	1.69 *** (1.39–2.04)	2.00 *** (1.44–2.82)	1.54 ** (1.16–2.04)
Moderate Depression ^b^	2.41 *** (2.00–2.92)	2.86 *** (2.13–3.85)	3.17 *** (2.54–3.96)	2.43 *** (1.97–3.00)	2.48 *** (1.75–3.53)	2.44 *** (1.84–3.26)
Moderately Severe Depression ^b^	3.06 *** (2.40–3.92)	3.19 *** (2.27–4.46)	3.84 *** (2.97–4.97)	3.03 *** (2.36–3.89)	4.31 *** (3.02–6.20)	3.40 *** (2.50–4.64)
Severe Depression ^b^	4.95 *** (3.46–7.24)	4.12 *** (2.74–6.13)	6.80 *** (4.95–9.38)	4.24 *** (3.07–5.92)	7.15 *** (4.84–10.64)	7.19 *** (5.09–10.20)
Model=	14.85, *p* = 0.06	2.52, *p* = 0.96	9.88, *p* = 0.27	5.34, *p* = 0.72	10.37, *p = 0*.24	5.36, *p* = 0.72
*n*	4095	4094	4088	3107	3111	3105
PTSD ^c^	2.65 *** (2.32–3.02)	2.96 *** (2.39–3.69)	3.61 *** (3.07–4.24)	2.42 *** (2.09–2.80	2.69 *** (2.17–3.37)	2.62 *** (2.17–3.17)
Model =	5.05, *p* = 0.75	10.51, *p* = 0.23	9.11, *p* = 0.33	7.48, *p* = 0.49	10.26, *p* = 0.25	8.20, *p* = 0.41
*n*	4096	4095	4089	3174	3178	3169
Moderate EE ^d^	2.34 *** (1.92–2.84)	3.15 *** (1.94–5.32)	2.54 *** (1.82–3.59)	1.79 *** (1.40–2.29)	1.91 * (1.15–3.29)	1.23 (0.81–1.89)
High EE ^d^	5.56 *** (4.67–6.63)	7.67 *** (5.01–12.41)	7.76 *** (5.82–10.56)	4.78 *** (3.87–5.94)	6.02 *** (3.94–9.68)	4.95 *** (3.56–7.06)
Model=	10.33, *p* = 0.24	6.11, *p* = 0.64	12.03, *p* = 0.15	6.05, *p* = 0.64	3.39, *p* = 0.91	8.30, *p* = 0.40
*n*	4055	4053	4048	3092	3096	3088
Moderate DP ^e^	1.98 *** (1.69–2.32)	3.52 *** (2.53–4.93)	2.46 *** (1.99–3.04)	2.04 *** (1.70–2.44)	1.52 ** (1.12–2.05)	1.91 *** (1.49–2.45)
High DP ^e^	4.24 *** (3.58–5.03)	9.33 *** (6.91–12.78)	5.14 *** (4.23–6.28)	4.32 *** (3.56–5.25)	5.19 *** (4.02–6.73)	4.83 *** (3.84–6.09)
Model=	10.26, *p* = 0.25	7.72, *p* = 0.46	10.77, *p* = 0.22	10.41, *p* = 0.24	6.07, *p* = 0.64	3.67, *p* = 0.89
*n*	4058	4057	4051	3093	3098	3091
Moderate PA ^f^	1.59 *** (1.36–1.85)	1.75 *** (1.29–2.39)	1.64 *** (1.34–2.00)	1.46 *** (1.22–1.74)	2.03 *** (1.47–2.84)	1.45 ** (1.13–1.87)
Low PA ^f^	2.99 *** (2.54–3.53)	4.40 *** (3.34–5.87)	2.59 *** (2.14–3.15)	2.92 *** (2.43–3.52)	5.53 *** (4.12–7.53)	3.07 *** (2.43–3.89)
Model=	5.83, *p* = 0.67	5.84, *p* = 0.66	6.49, *p* = 0.59	7.50, *p* = 0.48	8.76, *p* = 0.36	9.80, *p* = 0.28
*n*	4014	4014	4007	3038	3042	3035

Note: Paired wise deletion resulted in the exclusion of 11–15% of the data from logistic regression analyses at each time point. PTSD, post-traumatic stress disorder; EE, emotional exhaustion; DP, depersonalization; PA, personal accomplishment. Safety grade (failing, poor and acceptable = 1, very good and excellent = 0), general quality of nursing care (poor and fair = 1, good and excellent = 0), and recommend to family and friends (definitely no and probably no = 1, probably yes and definitely yes = 0) were reverse-scored; ^a^ reference group = no anxiety, ^b^ reference group = no depression, ^c^ reference group = no PTSD, ^d^ reference group = low EE, ^e^ reference group = low DP, ^f^ reference group= high PA. The models were adjusted for individual and workplace characteristics. *** *p*< 0.001, ** *p* < 0.01, * *p* < 0.05.

## Data Availability

Study data can be obtained from the principal investigator upon reasonable request.

## Supplementary Materials

The following supporting information can be downloaded at: https://www.mdpi.com/article/10.3390/healthcare10020314/s1. Table S1: The proportion of various severities of mental health symptoms before and during COVID-19; Figure S1: A visual depiction of odd ratios representing the relationship between anxiety and quality safety outcomes before and during COVID-19; Figure S2: A visual depiction of odd ratios representing the relationship between depression and quality safety outcomes before and during COVID-19; Figure S3: A visual depiction of odd ratios representing the relationship between PTSD and quality safety outcomes before and during COVID-19; Figure S4: A visual depiction of odd ratios representing the relationship between emotional exhaustion and quality safety outcomes before and during COVID-19; Figure S5: A visual depiction of odd ratios representing the relationship between depersonalization and quality safety outcomes before and during COVID-19; Figure S6: A visual depiction of odd ratios representing the relationship between personal accomplishment and quality safety outcomes before and during COVID-19.
