# Supplementary material for: The Association between Mental Health Symptoms and Quality and Safety of Patient Care before and during COVID-19 among Canadian Nurses
Source: Healthcare (Basel). 2022 Feb 7;10(2):314. doi: 10.3390/healthcare10020314 (PMC8871834; doi:10.3390/healthcare10020314)
Supplement: Supplementary file 1 [file healthcare-10-00314-s001.zip › healthcare-1543502-supplementary.pdf]

Table S1. The proportion of various severities of mental health symptoms before and during COVID-19

	n (%)	
	**Pre-COVID-19**	**During COVID-19**
No Anxiety	1621 (37.7)	878 (26.5)
Mild Anxiety	1474 (34.3)	1174 (35.5)
Moderate Anxiety	690 (16.1)	664 (20.1)
Severe Anxiety	510 (11.9)	594 (17.9)
N	4295	3310
No Depression	1647 (38.4)	922 (28.0)
Mild Depression	1321 (30.8)	1004 (30.5)
Moderate Depression	712 (16.6)	704 (21.4)
Moderately Severe Depression	409 (9.5)	428 (13.0)
Severe Depression	205 (4.8)	231 (7.0)
N	4294	3289
No PTSD	2259 (52.3)	1885 (52.6)
PTSD	2062 (47.7)	1700 (47.4)
N	4321	3585
Low EE	894 (21.5)	584 (18.3)
Moderate EE	951 (22.8)	676 (21.1)
High EE	2320 (55.7)	1939 (60.6)
N	4165	3199
Low DP	1787 (42.9)	1531 (47.9)
Moderate DP	1104 (26.5)	799 (25.0)
High DP	1276 (30.6)	868 (27.1)
N	4167	3198
Low PA	1329 (32.2)	1031 (32.8)
Moderate PA	1427 (34.6)	1046 (33.3)
High PA	1367 (33.2)	1064 (33.9)
N	4123	3141

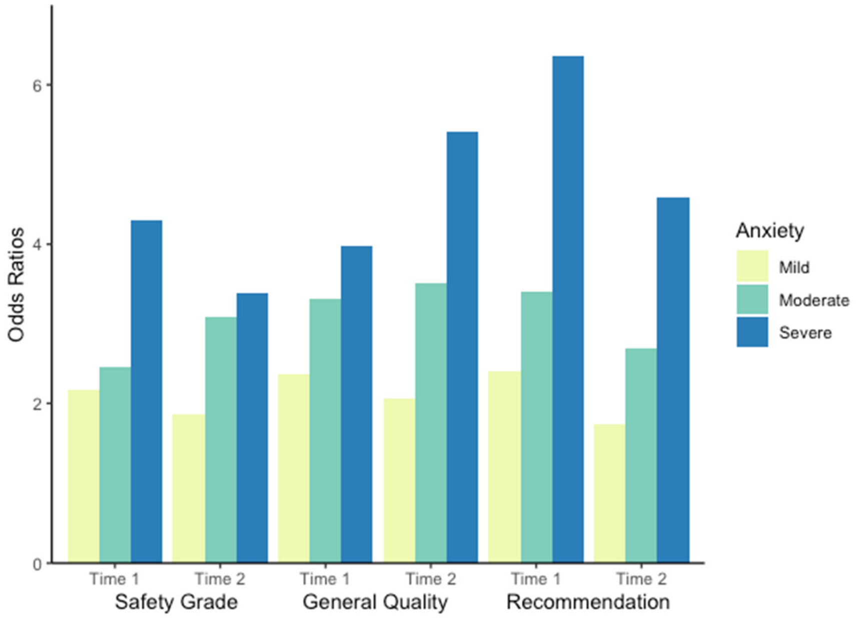

Figure S1. A visual depiction of odds ratios representing the relationship between anxiety and quality safety outcomes before and during COVID-19

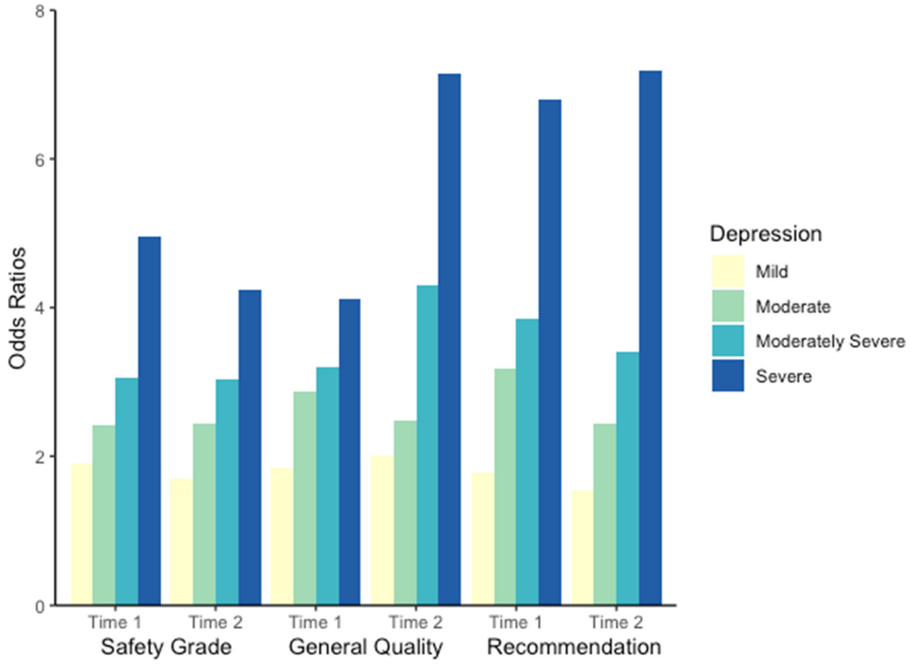

Figure S2. A visual depiction of odds ratios representing the relationship between depression and quality safety outcomes before and during COVID-19

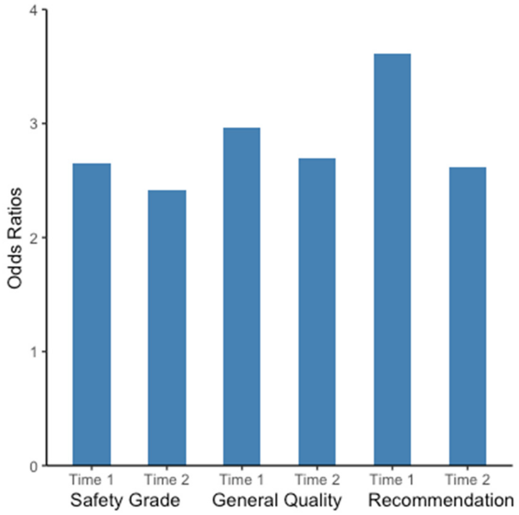

Figure S3. A visual depiction of odds ratios representing the relationship between PTSD and quality safety outcomes before and during COVID-19

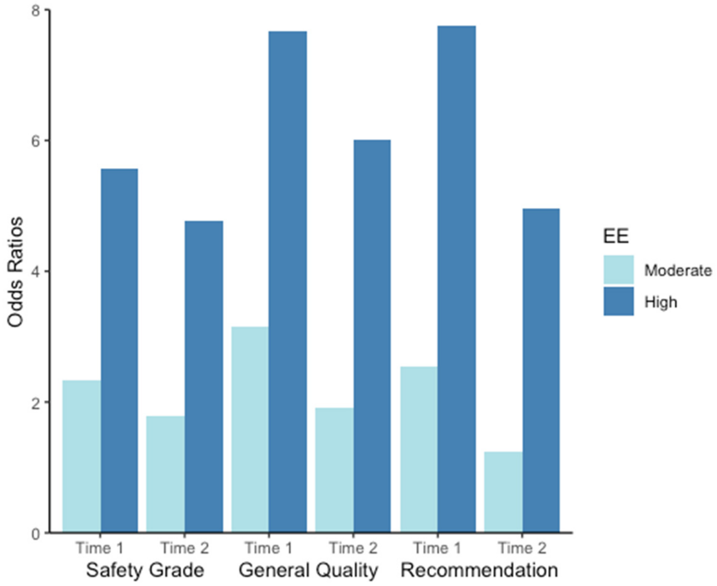

Figure S4. A visual depiction of odds ratios representing the relationship between emotional exhaustion and quality safety outcomes before and during COVID-19

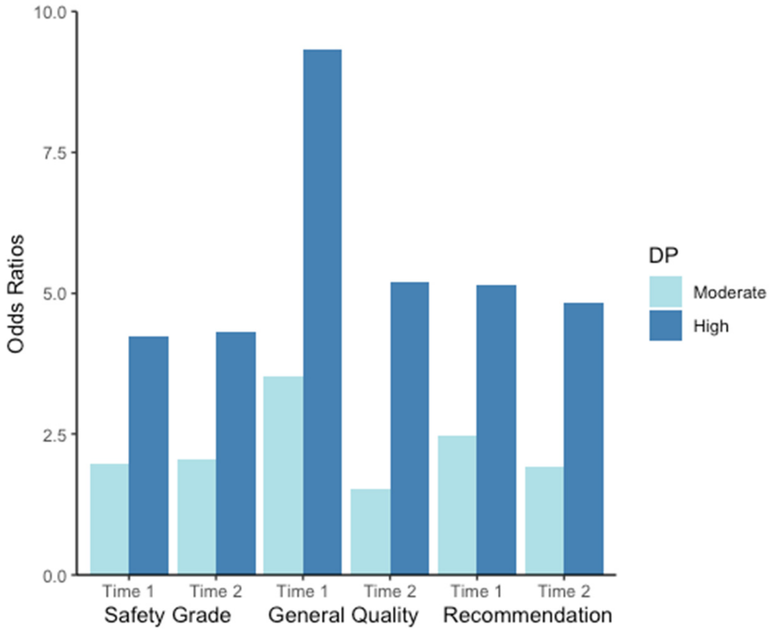

Figure S5. A visual depiction of odds ratios representing the relationship between depersonalization and quality safety outcomes before and during COVID-19

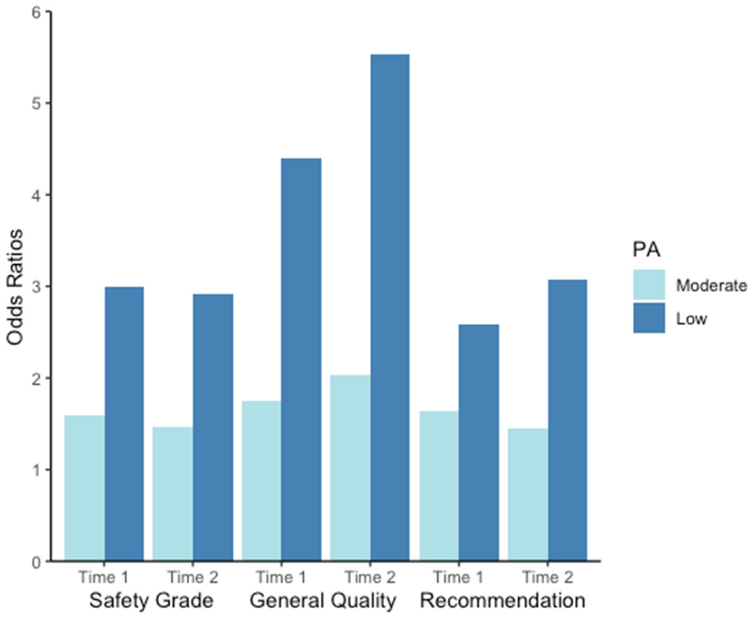

Figure S6. A visual depiction of odds ratios representing the relationship between personal accomplishment and quality safety outcomes before and during COVID-19
